# Investigation of the effects of different beverages on color change, surface roughness, and bacterial adhesion of nanohybrid and microhybrid composites

**DOI:** 10.1186/s12903-026-09076-x

**Published:** 2026-06-30

**Authors:** Ebru Yılmaz, Onur Akkurt, Nilgün Akgül, İlknur Kaleli, Mehmet Atça

**Affiliations:** 1 Department of Restorative Dentistry, Faculty of Dentistry, Pamukkale University, Denizli, 20160 Türkiye; 2 Department of Medical Microbiology, Faculty of Medicine, Pamukkale University, Denizli, 20160 Türkiye

**Keywords:** Composite resins, Color stability, Surface roughness, Bacterial adhesion, *Streptococcus mutans*

## Abstract

**Objective:**

This study aimed to analyze the impact of various beverages on the color change, surface roughness, and bacterial adhesion of nanohybrid and microhybrid composites.

**Materials and methods:**

Four resin composites with different filler characteristics were evaluated: two microhybrid composites (Dr. Müller Rhein [DRM] and Herculite Classic [HCM]) and two nanohybrid composites (Dr. Müller Kaiser [DRN] and Clearfil Majesty Esthetics [CME]). Each resin composite group was divided into four subgroups (*n* = 12) for immersion in beverages (control: distilled water; coffee, cola, and red wine). Initial color and surface roughness were measured, and measurements were repeated after 12 days of immersion. Saliva was applied to the sterilized sample surfaces to stimulate pellicle formation, and the samples were incubated. To evaluate bacterial adhesion, colony counts of *Streptococcus mutans* (ATCC 25175) were reported as CFU/ml. Additionally, one sample from each subgroup was cultured for scanning electron microscopy. Two-way analysis of variance was used to compare variables with normal distributions across resin composites and beverages; pairwise comparisons were performed using the Tukey test. Two-way robust ANOVA was used for variables that were not normally distributed, and pairwise comparisons were performed using the robust t-test with Holm’s correction. Results for quantitative variables are presented as mean ± standard deviation and median (minimum: maximum). The significance level was set at *p* < 0.05.

**Results:**

Dr. Müller Kaiser and Clearfil Majesty Esthetics were the most resistant to discoloration (*p* < 0.05). The highest surface roughness and bacterial adhesion were observed in the microhybrid composites Dr. Müller Rhein and Herculite Classic, while the lowest bacterial adhesion was observed in the nanohybrid composites (*p* < 0.05). Red wine and coffee exhibited the highest discoloration values, with no statistically significant difference between them (*p* > 0.05), whereas cola showed the greatest effect on surface roughness.

**Conclusions:**

Exposure of resin composites to various beverages can negatively affect surface properties, color stability, and promote biofilm formation. These findings provide preliminary insight into the effects of commonly consumed beverages on resin composites under controlled laboratory conditions. However, further in vivo and clinically simulated studies are required before definitive clinical recommendations can be made.

**Clinical significance:**

Considering patients’ dietary habits during material selection may improve long-term esthetic and biological outcomes.

## Introduction

Resin composites (RCs) are frequently preferred for the rehabilitation of lost tooth tissue due to their minimally invasive application, low cost, suitable mechanical properties, and improved aesthetic performance, enabled by recent technological advances [1–8]. In recent years, changes in the size, distribution, and type of particles in RCs have improved the mechanical strength and optical performance of these materials [2]. RCs can be classified based on the inorganic filler particles, which are available in different sizes and types, ranging from macro to nano scale. The mechanical and chemical properties of RCs depend on several factors, including the amount, type, shape, and distribution of fillers, the nature of the resin matrix, the interaction between fillers and matrix, and the finishing and polishing techniques used during restoration [2, 9]. Nano-filled and nanohybrid RCs have superior optical properties and translucency, and their improved polishability has been associated with the incorporation of smaller-sized nanofillers [10]. The introduction of these materials into clinical use has improved the surface quality and texture of restorations, enabling a more natural aesthetic appearance [11].

The long-term clinical performance of RCs has become controversial because chemical, mechanical, and thermal factors affect color stability, surface roughness, marginal integrity, and abrasion resistance [1, 2, 9, 12]. Color changes in composite restorations can be induced by intrinsic or extrinsic factors [13]. Intrinsic discoloration results from changes in the material’s chemical structure [14]. This can be caused by the infiltration of unreacted monomers due to hydrolysis and by the presence of photo-initiator components that are not fully utilized during light curing. Extrinsic factors include plaque and surface stain accumulation, surface degradation, and limited penetration and adhesion of staining agents to or near the surface of the RC [1, 11, 14, 15]. It has been reported that cola, fruit juice, red wine, tea, and coffee can lead to frequent discoloration between RC restorations and teeth [1, 4, 9, 16]. High consumption of these beverages can cause varying degrees of increased roughness and dulling on restoration surfaces; consequently, plaque accumulation and discoloration tendencies increase, optical properties are negatively affected, water absorption increases, and the integrity of restorations may be compromised [1, 3, 6, 9, 12, 14, 17].

Several studies have investigated biofilm formation on RCs [13, 16, 18–20]. Biofilms adhering to RCs contain microorganisms such as streptococci and lactobacilli that can survive in highly acidic environments [18]. It has been reported that the *Streptococcus mutans (S. mutans)* count in biofilms plays a key role in determining cariogenic potential [8, 13, 21]. The marginal regions of dental restorations are particularly susceptible to bacterial adhesion [22], and adhesion can be influenced by surface roughness, free surface energy, antibacterial agents, and the type and size of the filler [8, 13, 18, 19, 23]. Biofilm formation on RC surfaces is clinically important because it may promote plaque accumulation, increase the risk of secondary caries, and compromise the long-term success of restorations. Therefore, evaluating bacterial adhesion after exposure to commonly consumed beverages provides clinically relevant insight into the biological performance of these materials.

Despite extensive research on resin composites, there remains a lack of comprehensive studies simultaneously evaluating color stability, surface roughness, and bacterial adhesion in relation to compositional differences between nanohybrid and microhybrid composites. The influence of these compositional differences on these parameters remains insufficiently understood. Therefore, this study aimed to address this gap by comparatively evaluating these properties under standardized conditions.

The null hypotheses were as follows: there would be no statistically significant differences between microhybrid and nanohybrid resin composites after immersion in different beverages in terms of (1) color change, (2) surface roughness, or (3) bacterial adhesion. In addition, beverage exposure would not significantly affect these optical, surface, and biological properties of the tested resin composites. Accordingly, this study aimed to evaluate the combined effects of resin composite type, particularly microhybrid versus nanohybrid filler characteristics, and beverage exposure on color stability, surface roughness, and *S. mutans* adhesion.

## Materials and methods

### Ethics approval and consent to participate

This study was approved by the Pamukkale University Non-Interventional Clinical Research Ethics Committee (Approval No: E-60116787-020-615617). The Declaration of Helsinki was provided to each investigator by the Pamukkale University Ethics Committee, and their consent was obtained; the study was conducted in accordance with the principles of the Declaration of Helsinki. In this study, a saliva sample belonging to one of the researchers was used, and informed consent was obtained. The use of a single saliva donor was intended to ensure experimental standardization and reduce inter-individual variability in pellicle formation; however, this approach may limit the biological representativeness of the acquired pellicle.

The following RC groups were tested in the study: Dr. Müller Rhein – microhybrid composite (DRM; MANI Medical Germany GmbH, Rosbach vor der Höhe, Germany), Herculite Classic – microhybrid composite (HCM; Kerr Corp., Orange, CA, USA), Dr. Müller Kaiser – nanohybrid composite (DRN; MANI Medical Germany GmbH, Rosbach vor der Höhe, Germany), and Clearfil Majesty Esthetics – nanohybrid composite (CME; Kuraray Europe GmbH, Hattersheim am Main, Germany). The properties of these RCs are presented in Table 1.

**Table 1:** Resin composites used and their properties

Resin Composite	Manufacturer	Type	Shade	Composition	Filler amount, wt%, vol%	Lot No
Dr. Müller Kaiser (DRN)	MANI Medical Germany GmbH, Rosbach vor der Höhe, Germany	Nanohybrid	A2	Glass powder, diurethane dimethacrylate, silicon dioxide, Bis-GMA, tetramethylene dimethacrylate, splitter polymerizate particle size range: 40 μm- 28 nm	83,5 / 66,5	2023007936
Herculite Classic (HCM)	Kerr Corp., Orange, CA, USA	Microhybrid	A2	Bis-GMA, TEGDMA, camphorquinone, amine-based initiators, iron oxide pigments, aluminoborosilicate glass, colloidal silica. particle size range: average 0,6 μm	79 / 59	6933034
Dr. Müller Rhein (DRM)	MANI Medical Germany GmbH, Rosbach vor der Höhe, Germany	Microhybrid	A2	Glass powder, diurethane dimethacrylate, silicon dioxide, Bis-GMA, tetramethylene dimethacrylate particle size range: 0,005 − 3,0 μm	75 / 53	2023007809
Clearfil Majesty Esthetics (CME)	Kuraray Europe GmbH, Hattersheim am Main, Germany	Nanohybrid	A2	Bis-GMA, silanated barium glass filler, pre-polymerized organic filler, hydrophobic aromatic dimethacrylate, hydrophobic aliphatic dimethacrylate dl-camphorquinone, accelerators, initiators, pigments particle size range: 0.7 μm − 20 nm	78 / 66	00034A

**Fig. 1:**
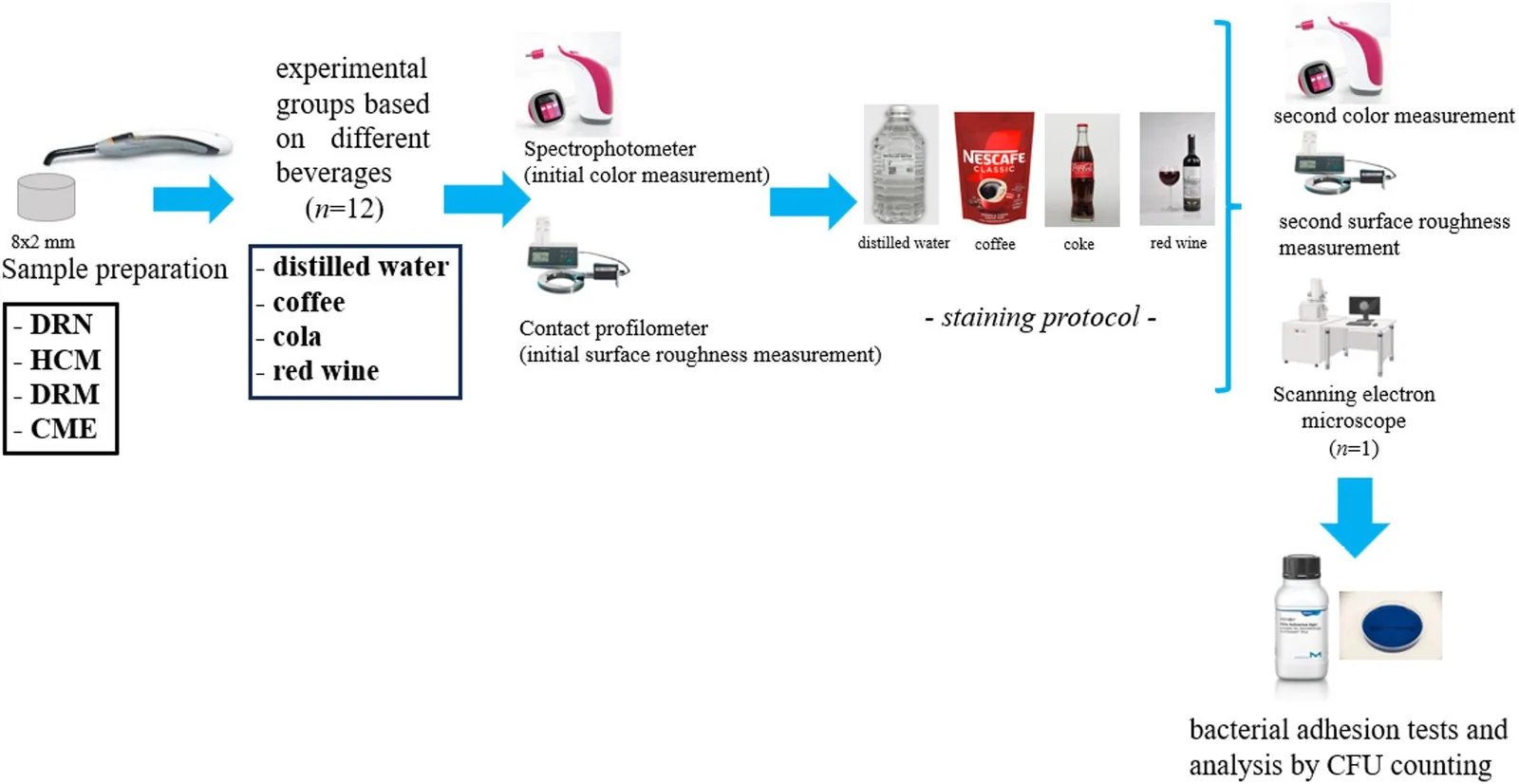
A schematic illustration of the study design and methodological workflow. DRN: Dr. Müller Kaiser Nanohybrid composite, HCM: Herculite Classic Microhybrid Composite, DRM: Dr. Müller Rhein Microhybrid composite, CME: Clearfil Majesty Esthetics

Information on the beverages (control group: distilled water, coffee, cola, and red wine) is presented in Table 2. The workflow outlining the materials and methods of the study is presented in Fig. 1.

**Table 2:** Beverages used in the study

Beverage	Manufacturer	Composition	pH
Distilled water	-	-	6.98
Coffee	Nestlé, Vevey, Switzerland	Instant coffee	5.7
Cola	The Coca Cola Company, Atlanta, USA	Carbonated water, sugar, caramelcolor, phosphoric acid, naturalflavors, caffeine	2.7
Red wine	Doluca Red, İstanbul, Türkiye	Sugar‑free red wine, alcohol content 12.5% Vol	4.05

### Sample size

To calculate the sample size, a power analysis using G*Power (G*Power Ver. 3.1.9.4, Heinrich-Heine-Universität Düsseldorf, Germany) yielded a minimum sample size of 172, given an effect size of 0.3, a 5% significance level, and a 95% confidence interval. The study was conducted on 192 samples. Furthermore, one sample from each subgroup was prepared for scanning electron microscopy (SEM). The sample size calculation was based on an overall effect size and was not performed separately for each outcome variable (Δ*E*_00_, Ra, and CFU).

### Preparation of the samples

A total of 192 samples, 12 per subgroup, were prepared in 8 × 2 mm cylindrical Teflon molds for color change, surface roughness, and bacterial adhesion experiments. After the RC was placed in the Teflon mold with a mouth spatula, the material was compressed between two Mylar strips and pressed with a glass coverslip to prevent air bubble formation and remove excess material. The samples were polymerized for 20 s with an LED light at a power density of 1200 mW/cm^2^ (Bluephase Powercure, Ivoclar Vivadent AG, Schaan, Liechtenstein).

The standard polishing protocol was applied to the polymerized samples using aluminum oxide discs (Sof-Lex; 3 M Company, St. Paul, MN, USA) ranging from coarse to super-fine grit. The samples were randomly divided into four subgroups based on the beverage in which they were immersed (control: distilled water; coffee; cola; and red wine). The coffee solution was prepared by dissolving 2 g of coffee powder (Nescafé Classic, Nestlé, Vevey, Switzerland) in 200 ml of boiled distilled water, following the manufacturer’s instructions. Distilled water, cola, and red wine were not processed. All immersion procedures were conducted at room temperature to ensure standardization across groups and to avoid variability related to temperature fluctuations; however, this condition does not fully replicate intraoral thermal variations. The beverages were changed daily to prevent microbial growth and to help maintain consistent exposure conditions. The samples were exposed to these beverages for 12 days as an accelerated aging protocol, which has been previously used to approximate long-term exposure (approximately corresponding to one year) in in vitro studies [1, 24]. This approach represents a simplified model and may not fully reflect real clinical exposure conditions. After immersion, the samples were washed with deionized water for 10 s and gently dried before measurement to avoid surface damage.

### Color measurement

The baseline color (T0) of the samples before immersion in the beverages was measured using a spectrophotometer (VITA Easyshade V, VITA Zahnfabrik, Bad Säckingen, Germany). The spectrophotometer was calibrated prior to each measurement session according to the manufacturer’s instructions. To ensure standardization, a gray background mimicking the oral environment was used, and the probe was positioned perpendicular to the sample surface using a standardized positioning approach. All measurements were performed by the same operator in a daylight-lit room at the same time of day to minimize operator- and environment-related variability. To improve repeatability and reduce measurement error, three consecutive measurements were obtained from the central region of each specimen, and the mean values were recorded as L*, a*, and b* coordinates. Although measurements were obtained from the central region to standardize positioning, this approach may not fully represent surface heterogeneity. Measurements were repeated at the end of the 12th day (T1) after immersion. The color difference (Δ*E*_00_) was calculated using the CIEDE2000 formula [24].

$$\:{\Delta\:}{E}_{00}=\sqrt{{\left(\frac{{\Delta\:}{L}^{{\prime\:}}}{{k}_{L}{S}_{L}}\right)}^{2}+{\left(\frac{{\Delta\:}{C}^{{\prime\:}}}{{k}_{C}{S}_{C}}\right)}^{2}+{\left(\frac{{\Delta\:}{H}^{{\prime\:}}}{{k}_{H}{S}_{H}}\right)}^{2}+{R}_{T}\frac{{\Delta\:}{C}^{{\prime\:}}}{{k}_{C}{S}_{C}}\frac{{\Delta\:}{H}^{{\prime\:}}}{{k}_{H}{S}_{H}}}$$

The threshold values for perceptibility and acceptability were set at 0.8 and 1.8, respectively [3, 25].

### Surface roughness measurement

Surface roughness (Ra) of the samples was measured before immersion (T0) with a contact profilometer (Perthometer M2, Mahr GmbH, Göttingen, Germany). The cutoff value for surface roughness was set to 0.25 mm, and the analysis length to 1.75 mm. Measurements were performed using a contact stylus under a constant applied force, as specified by the manufacturer. To improve repeatability and account for surface heterogeneity, three measurements were obtained from different regions of each specimen, and the mean Ra value was recorded. Before each use, the device was calibrated with a reference block with a known Ra of 3.22 μm. Surface roughness measurements were repeated after the samples were immersed in the respective beverage groups for 12 days (T1).

### SEM analysis

One representative specimen from each subgroup was evaluated after 12 days of immersion in the test beverages. SEM images were obtained using a scanning electron microscope (Quanta 650 Field Emission, FEI Company, Hillsboro, USA). For SEM analysis, the samples were coated with gold on carbon tape under high vacuum to provide surface conductivity. SEM observations were conducted at 15000x magnification to obtain 20 kV images. Therefore, SEM findings were considered qualitative and illustrative rather than suitable for statistical generalization. No quantitative analysis was performed based on SEM observations.

### Bacterial adhesion tests and analysis by CFU counting

The study samples were packaged by RC subgroup and sterilized with ethylene oxide [22]. The author’s unstimulated saliva sample was centrifuged at 15,000 g for 15 min at 4 °C. The supernatant was filtered through a 0.22 μm filter and stored at -20 °C. The test was performed in sterilized 24-well plates. 500 µl of saliva was added to each composite disc to stimulate pellicle formation, and the plates were incubated at 37 °C for 1 h, as previously described for saliva-coated *S. mutans* adhesion on resin composite surfaces [8]. The pellicle-coated discs were washed with 2 ml of phosphate-buffered saline (PBS) and transferred to new 24-well plates. 1.6 ml of brain heart infusion broth (BHI) enriched with 5% sucrose was used to coat the samples. Samples were inoculated with 200 µl of *S. mutans* ATCC 25,175 bacterial suspension [final concentration of 1 × 10⁸ colony-forming units (CFU)/ml]. The bacterial inoculum was standardized by adjusting the turbidity of the suspension to match the 0.5 McFarland standard prior to dilution, ensuring reproducibility across all experimental groups. After incubation at 37 °C for 24 h, the discs were washed three times with 2 ml of physiological saline to remove non-adherent bacteria and placed in sterile glass tubes containing 1 ml of physiological saline per disc. The discs were vortexed for 60 s to collect the adherent biofilm. The bacteria were then transferred to sterile physiological saline, 1:10 diluted for counting, and 50 µl of suspension from both the undiluted and 1:10 diluted tubes were inoculated onto Mitis Salivarius Bacitracin Agar (MSB) medium and spread. All media were incubated anaerobically at 37 °C for 24 h to allow colony formation. Anaerobic conditions were used to minimize oxidative stress and support optimal growth of *S. mutans*. All samples from all experimental groups were evaluated under identical conditions and within the same time frame to ensure consistency across bacterial adhesion assessment. The number of colonies formed was counted in CFU/ml.

### Statistical analysis

Data were analyzed using IBM SPSS V23 (IBM Corp., Armonk, NY, USA) and Jamovi (version 2.3.28; The Jamovi Project, Sydney, Australia). The Shapiro-Wilk test was used to assess normality. Two-way analysis of variance was used to compare normally distributed variables by RC and beverage. Pairwise comparisons were performed using the Tukey test, and two-way robust ANOVA was used to compare variables that were not normally distributed. Pairwise comparisons were conducted using the robust t-test with Holm correction. For normally distributed variables, the Pearson correlation coefficient was used to assess correlations within each RC and beverage, while Spearman’s rho was used for variables that were not normally distributed. The correlation analyses were planned a priori and were limited to two biologically and clinically relevant variable pairs: surface roughness and bacterial adhesion (Ra vs. CFU), and surface roughness and color change (Ra vs. Δ*E*_00_). These correlations were selected because surface roughness may influence bacterial retention and may also be associated with surface degradation and discoloration. A direct correlation between color change and bacterial adhesion (Δ*E*_00_ vs. CFU) was not analyzed because color change primarily reflects optical alterations related to pigment absorption/adsorption and material discoloration, whereas bacterial adhesion is mainly influenced by surface topography, pellicle characteristics, and surface-related physicochemical properties. Results for quantitative variables are presented as mean ± standard deviation and median (minimum: maximum). Due to the multifactorial design of the study, statistical analyses were conducted separately for each outcome variable. The significance level was set at *p* < 0.05.

## Results

Table 3 compares the mean Δ*E*_00_ values across RCs and beverages. For the RC, beverage, and RC × beverage interaction groups, there were statistically significant differences in the mean Δ*E*_00_ values (*p* < 0.001). The significant RC × beverage interaction indicated that the discoloration response to beverages differed among the tested RC materials. In particular, higher Δ*E*_00_ values were observed in the DRM and HCM groups after immersion in coffee and red wine, whereas DRN and CME showed comparatively lower Δ*E*_00_ values under the same beverage conditions. Distilled water produced the lowest Δ*E*_00_ values across all RC materials, while cola generally resulted in lower discoloration than coffee and red wine. The highest mean was observed in the DRM composite, followed by HCM, CME, and DRN composites. No statistically significant difference was found between DRM and HCM, or between CME and DRN composites. Within the beverage groups, the mean Δ*E*_00_ values were ordered as red wine > coffee > cola > distilled water. No statistically significant difference was found between coffee and red wine despite both groups demonstrating the highest discoloration values, while statistically significant differences were observed among the remaining groups (*p* < 0.001).

**Table 3:** Comparison of mean Δ*E*_00_ values according to resin composites and beverages

	Beverages				Total		F	*p*	$\eta^2$
	Distilled water	Coffee	Cola	Red wine					
RCs									
DRN	0.8 ± 0.51^F^	3.13 ± 1.05^CDE^	2.41 ± 0.88^E^	3.54 ± 0.74^BC^	2.47 ± 1.32^b^	**RC**	31.170	<0.001	0.347
HCM	1.4 ± 0.58^F^	4.32 ± 0.78^AB^	3.01 ± 0.52^CDE^	4.69 ± 0.72^A^	3.36 ± 1.45^a^	**Beverage**	287.920	<0.001	0.831
DRM	1.11 ± 0.28^F^	4.7 ± 0.54^A^	3.25 ± 0.36^CD^	4.62 ± 0.42^A^	3.42 ± 1.52^a^	**RC × Beverage**	2.110	0.031	0.097
CME	0.94 ± 0.21^F^	3.7 ± 0.33^BC^	2.51 ± 0.28^DE^	3.76 ± 0.24^BC^	2.72 ± 1.19^b^				
Total	1.06 ± 0.47^c^	3.96 ± 0.93^a^	2.8 ± 0.65^b^	4.15 ± 0.75^a^	2.99 ± 1.43				

^a-c^ There is no difference between main effects sharing the same letter ; ^A-F^ There is no difference between interaction groups with the same letter

*F* two-way analysis of variance test statistic, $\eta^2$ Partial Eta Squared, *R*^2^=%84.23, *RCs* Resin Composites, *DRN *Dr. Müller Kaiser Nanohybrid composite, *HCM *Herculite Classic Microhybrid composite, *DRM* Dr. Müller Rhein Microhybrid composite, *CME *Clearfil Majesty Esthetics

Table 4 compares the mean Ra values across RCs and beverages. Statistically significant differences were observed in the mean Ra values based on RC type and beverage (*p* < 0.001), but no statistically significant difference was observed for the RC type × beverage interaction. This non-significant interaction indicates that the effect of beverage exposure on Ra did not differ significantly among the tested RC materials, unlike the interaction patterns observed for Δ*E*_00_ and bacterial adhesion. The highest mean Ra was observed in DRM and HCM composites, with no statistically significant difference between them. When evaluated by beverage group, the lowest mean Ra was observed in distilled water, and the highest was observed in the cola, with statistically significant differences among all beverage groups (*p* < 0.001). However, all mean Ra values were below 0.2 μm, the clinically relevant threshold for plaque retention.

**Table 4:** Comparison of mean Ra values according to resin composites and beverages

	Beverages				Total		F	*p*	$\eta^2$
	Distilled water	Coffee	Cola	Red wine					
RCs									
DRN	0.1 ± 0.01	0.15 ± 0.02	0.18 ± 0.01	0.16 ± 0.01	0.15 ± 0.03^b^	**RC**	15.060	<0.001	0.204
HCM	0.12 ± 0.02	0.17 ± 0.01	0.19 ± 0.02	0.19 ± 0.02	0.17 ± 0.04^a^	**Beverage**	157.910	<0.001	0.729
DRM	0.13 ± 0.01	0.17 ± 0.02	0.19 ± 0.01	0.18 ± 0.03	0.17 ± 0.03^a^	**RC×Beverage**	1.180	0.309	0.057
CME	0.11 ± 0.01	0.14 ± 0.03	0.19 ± 0.02	0.17 ± 0.02	0.15 ± 0.04^b^				
Total	0.11 ± 0.02^d^	0.16 ± 0.02^c^	0.19 ± 0.02^a^	0.18 ± 0.02^b^	0.16 ± 0.04				

^a-d^ There is no difference between main effects sharing the same letter

*F* two-way analysis of variance test statistic, $\eta^2$ Partial Eta Squared, *R*^2^=%95.6, *RCs *Resin Composites, *DRN *Dr. Müller Kaiser Nanohybrid composite, *HCM *Herculite Classic Microhybrid composite, *DRM *Dr. Müller Rhein Microhybrid composite, *CME *Clearfil Majesty Esthetics

**Fig. 2:**
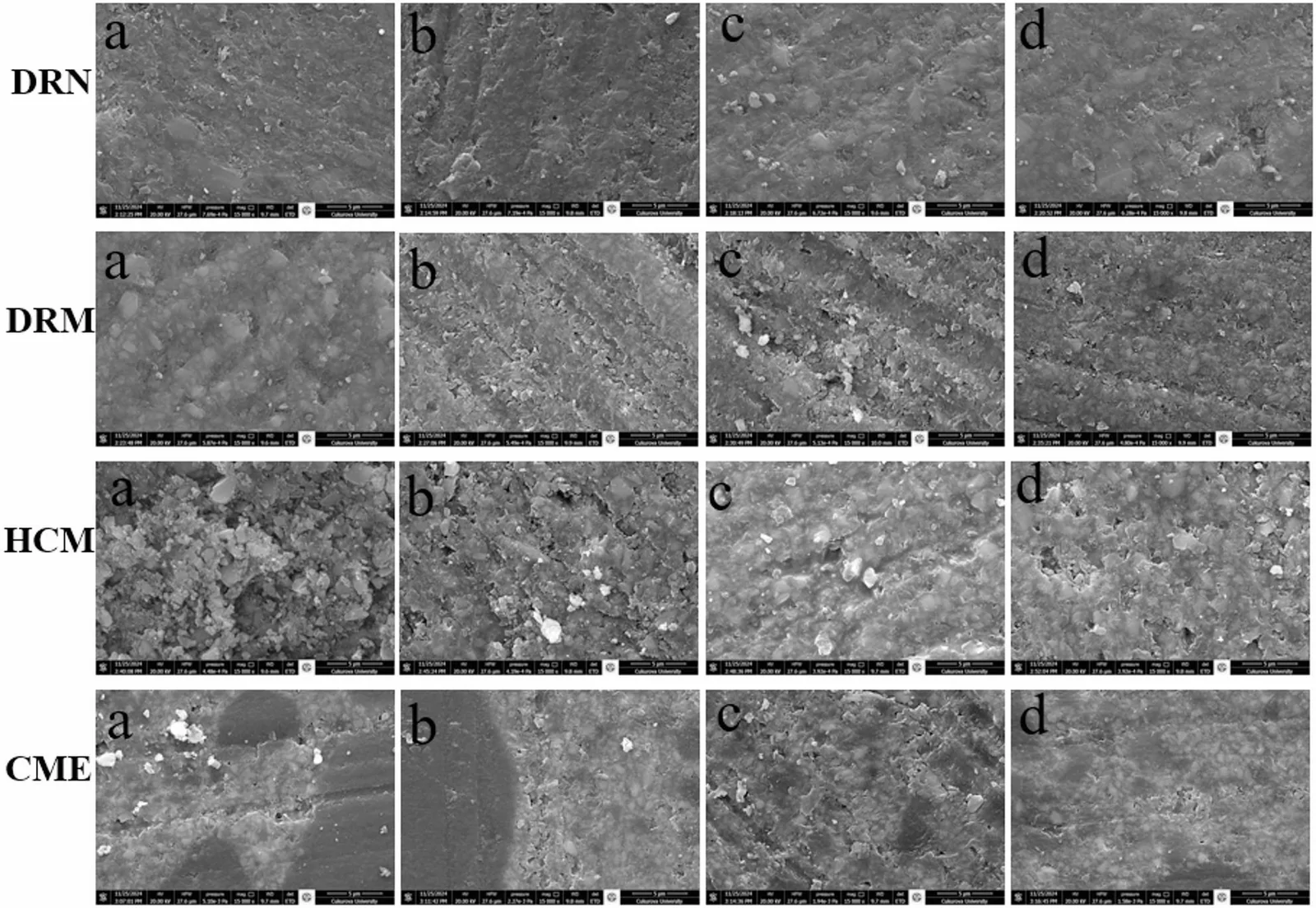
SEM images at 15000× magnification after RCs were immersed in different beverages: **a**) distilled water, **b**) coffee, **c**) cola, **d**) red wine; DRN: Dr. Müller Kaiser Nanohybrid composite, DRM: Dr. Müller Rhein Microhybrid composite, HCM: Herculite Classic Microhybrid Composite, CME: Clearfil Majesty Esthetics

Representative SEM images in Fig. 2 showed observable differences in surface morphology among the RC and beverage subgroups. Specimens immersed in distilled water generally exhibited relatively smoother and more homogeneous surfaces, whereas specimens exposed to coffee, cola, and red wine showed more irregular surface features. Cola- and red wine-exposed specimens displayed more pronounced surface irregularities and granular surface appearances. Microhybrid composites, particularly DRM and HCM, exhibited more evident surface irregularities than the nanohybrid composites, while DRN and CME showed comparatively smoother surface appearances. These SEM observations were qualitative and were used to support the profilometric findings rather than for statistical comparison.

Table 5 compares the median *S. mutans* adhesion across RCs and beverages. The median *S. mutans* adhesion showed statistically significant differences across RC type, beverage, and interaction groups (*p* < 0.001). The significant RC × beverage interaction indicated that the effect of beverage exposure on *S. mutans* adhesion differed among the tested RC materials. In the distilled water groups, all RC materials showed relatively low adhesion values and shared the same interaction group label. After beverage exposure, however, the adhesion pattern varied according to the material. The highest adhesion values were observed in the DRM-cola group, followed by HCM-red wine, DRM-coffee, and HCM-cola groups. In contrast, CME generally showed lower adhesion values after exposure to coffee and cola compared with the microhybrid composites. Although coffee, cola, and red wine did not differ significantly as main beverage effects, the significant interaction indicated that their effects were not uniform across the different RC materials. The highest median values were observed in the DRM and HCM composites, with no statistically significant difference between them. However, statistically significant differences were observed between these groups and the others (*p* < 0.001). The lowest median value was observed in the CME composite, with a statistically significant difference from the other groups. When evaluated by beverage group, the lowest median was observed in distilled water, with a statistically significant difference from the other groups (*p* < 0.001). No statistically significant difference was found among the coffee, cola, and red wine samples. The clinical relevance of these differences should be interpreted with caution.

**Table 5:** Comparison of median *S. mutans* adhesion values according to resin composites and beverages

	Beverages				Total		Q	*p*	$\eta^2$
	Distilled water	Coffee	Cola	Red wine					
RCs									
DRN	12275(8120: 29600)^A;^	54850(44250: 83900)^B^	50375(36200: 89900)^BC^	41850(28450: 72900)^CD^	45375(8120: 89900)^c^	**RC**	20.2	<0.001	0.256
HCM	13825(5420: 19350)^A^	69025(53200: 119800)^EF^	75975(53200: 119500)^EG^	84000(58200: 129800)^EG^	67725(5420: 129800)^b^	**Beverage**	120.9	<0.001	0.673
DRM	16025(8150: 42800)^A^	75875(49200: 106900)^EG^	87175(65800: 148900)^G^	59825(40200: 86900)^BF^	64775(8150: 148900)b	**RC × Beverage**	66.2	<0.001	0.772
CME	10350(5200: 27000)^A^	42550(29600: 62700)^CD^	35000(23100: 62500)^D^	47450(30800: 86900)^BC^	35875(5200: 86900)^a^				
Total	13075(5200: 42800)^b^	59550(29600: 119800)^a^	63600(23100: 148900)^a^	57450(28450: 129800)^a^	49750(5200: 148900)				

Median (minimum: maximum); ^a-c^ No difference among primary effects sharing the same letter; ^A-G^:No difference among interaction groups sharing the same letter

*Q* two-way robust ANOVA test statistic, $\eta^2$ Partial Eta Squared, *RCs *Resin Composites, *DRN *Dr. Müller Kaiser Nanohybrid composite, *HCM *Herculite Classic Microhybrid composite, *DRM *Dr. Müller Rhein Microhybrid composite, *CME *Clearfil Majesty Esthetics

Table 6 presents correlations between surface roughness, ∆*E*_00_, and bacterial adhesion across beverage subgroups within each RC. In the DRN-distilled water (*r* = 0.762; *p* = 0.004), DRM-coffee (*r* = 0.703; *p* = 0.011), DRM-cola (*r* = 0.61; *p* = 0.035), DRM-red wine (*r* = 0.858; *p* = 0), CME-coffee (*r* = 0.733; *p* = 0.007), CME-cola (*r* = 0.708; *p* = 0.01), and CME-red wine (*r* = 0.579; *p* = 0.048) subgroups, surface roughness and bacterial adhesion were significantly correlated. In the HCM-cola (*r* = 0.705; *p* = 0.01) and DRM-red wine (*r* = 0.614; *p* = 0.034) subgroups, surface roughness and ∆*E*_00_ showed a statistically significant positive correlation.

**Table 6:** Correlation of surface roughness with color change and bacterial adhesion

RCs	Beverages	Variable	Surface roughness	
			*r*	*p*
DRN	Distilled water	Color change	0.139^x^	0.667
		Bacterial adhesion	0.762^y^	0.004
	Coffee	Color change	0.059^x^	0.856
		Bacterial adhesion	-0.137^x^	0.672
	Cola	Color change	0.500^x^	0.098
		Bacterial adhesion	0.555^x^	0.061
	Red wine	Color change	0.070^x^	0.829
		Bacterial adhesion	-0.306^x^	0.334
HCM	Distilled water	Color change	0.028^x^	0.930
		Bacterial adhesion	0.470^x^	0.123
	Coffee	Color change	0.432^x^	0.161
		Bacterial adhesion	0.336^x^	0.286
	Cola	Color change	0.705^x^	0.010
		Bacterial adhesion	0.418^x^	0.177
	Red wine	Color change	0.298^x^	0.346
		Bacterial adhesion	0.542^x^	0.069
DRM	Distilled water	Color change	0.248^x^	0.437
		Bacterial adhesion	0.390^x^	0.210
	Coffee	Color change	0.282^x^	0.375
		Bacterial adhesion	0.703^x^	0.011
	Cola	Color change	-0.427^x^	0.166
		Bacterial adhesion	0.610^x^	0.035
	Red wine	Color change	0.614^x^	0.034
		Bacterial adhesion	0.858^x^	< 0.001
CME	Distilled water	Color change	0.449^x^	0.143
		Bacterial adhesion	0.559^x^	0.059
	Coffee	Color change	0.056^x^	0.862
		Bacterial adhesion	0.733^x^	0.007
	Cola	Color change	-0.005^x^	0.987
		Bacterial adhesion	0.708^x^	0.010
	Red wine	Color change	0.264^x^	0.408
		Bacterial adhesion	0.579^x^	0.048

^x^ Pearson correlation coefficient; ^y^ Spearman’s rho correlation coefficient

*RCs* Resin Composites, *DRN *Dr. Müller Kaiser Nanohybrid composite, *HCM *Herculite Classic Microhybrid composite, *DRM *Dr. Müller Rhein Microhybrid composite, *CME *Clearfil Majesty Esthetics

## Discussion

In clinical applications, matching the color of RCs to the surrounding tooth tissue and maintaining that color over time are fundamental to achieving aesthetically satisfactory results [3]. The present study investigated the effects of various beverages (distilled water, coffee, cola, and red wine) on color change, surface roughness, and bacterial adhesion of nanohybrid and microhybrid RCs. The findings showed that ∆*E*_00_ caused by coffee, cola, and red wine in all RCs exceeded the clinically acceptable threshold, with the greatest changes induced by red wine and coffee, which differed statistically from the other groups (*p* < 0.001). Thus, the first null hypothesis, that there would be no statistically significant differences between microhybrid and nanohybrid resin composites after beverage immersion in terms of color change and that beverage exposure would not significantly affect color change, was rejected. These findings suggest that beverage type played a major role in discoloration, with red wine and coffee showing the greatest staining potential, particularly in microhybrid RCs.

Clinically perceptible and acceptable thresholds are commonly used to evaluate color change. In our study, the clinically perceptible threshold was 0.8, and the acceptable threshold was 1.8 [26]. The data showed that ∆*E*_00_ exceeded the clinically perceptible value in the distilled water group and the acceptable threshold in the other beverage groups. This indicates that the current study aligns with previous reports [26, 27].

The discoloration observed over time in RC samples immersed in distilled water is attributed to the polymer network swelling in water, which reduces frictional forces between polymer chains. Furthermore, as RCs absorb water, they can also absorb other liquids that cause discoloration, such as coffee, cola, and red wine [2]. The fact that Δ*E*_00_ values in the distilled water group exceeded the perceptibility threshold indicates that perceptible color changes may occur even under aqueous conditions without chromogenic exposure. This finding suggests that the baseline color stability of the tested RC materials may be affected by water sorption and hydrolytic changes alone, independent of external staining agents. Therefore, water-mediated color alteration should be considered when interpreting the discoloration potential of RCs.

Color change in RCs is mainly associated with the absorption and adsorption of chromogenic pigments, which can penetrate the organic matrix due to their chemical affinity with the polymer phase [2]. The study’s findings demonstrated that cola, despite being more acidic than coffee, had a lesser effect on color change. This is consistent with previous reports in the literature [2, 11, 26, 28]. This finding may be explained by the absence of strong chromogenic agents in cola, despite its low pH, which primarily affects surface roughness rather than discoloration. Although cola’s low pH may enhance surface solubility and soften the polymer matrix, the limited color change observed may also be attributed to the absence of yellow colorants in its composition [28].

Tanthanuch et al. [29] reported that discoloration of RCs from exposure to tea, wine, and coffee exceeded the clinically perceptible threshold. Similarly, Da Silva et al. reported that coffee induces greater discoloration than less chromogenic beverages [24].

Materials with higher TEGDMA content than other RCs exhibit greater water absorption due to TEGDMA’s pronounced hydrophilic nature. This increases free volume in the polymer network, reducing color stability and accelerating water diffusion [1, 30, 31]. This mechanism may partly explain the higher Δ*E*_00_ observed in HCM, which contains TEGDMA, compared with DRN and CME, which do not contain TEGDMA. However, DRM showed the highest Δ*E*_00_ despite not containing TEGDMA, indicating that discoloration cannot be explained by TEGDMA content alone. The greater discoloration of DRM may be related to other material-dependent factors, such as its lower filler loading compared with DRN, differences in filler particle size distribution, filler-matrix interfacial characteristics, and the composition or hydrophilicity of the resin matrix. These factors may increase water sorption, matrix plasticization, and pigment penetration, thereby reducing color stability. In addition, larger or more heterogeneous filler particles may negatively affect color stability, which may partly explain the difference between DRN and DRM despite their broadly similar composition [32].

Increasing the matrix ratio in RCs increases water absorption and weakens bonds between the resin matrix and filler particles. This can lead to microcracks in the resin matrix and void formation at the filler-matrix interface due to swelling and plasticization. These structural changes can facilitate the penetration of external pigments into the material, thereby negatively affecting the color stability of restorations [11]. Although the filler ratios of DRM, HCM, and CME are similar, DRN, with the highest filler ratio (wt 83.5%), is the most color-stable material in terms of coloration.

The significant RC × beverage interactions observed for both Δ*E*_00_ and *S. mutans* adhesion indicate that the effects of beverage exposure were not uniform across the tested RC materials. For color change, the highest Δ*E*_00_ values were observed in the microhybrid composites, particularly DRM and HCM, after exposure to coffee and red wine, whereas the nanohybrid composites, especially DRN and CME, showed comparatively lower discoloration under the same conditions. This pattern suggests that the staining potential of chromogenic beverages may be more pronounced in materials with microhybrid filler characteristics, possibly due to differences in filler size, filler-matrix interface, resin matrix composition, and water sorption behavior. Similarly, the interaction pattern for bacterial adhesion showed that beverage-related changes were material-dependent, with the highest adhesion observed in the DRM-cola subgroup and high values also observed in HCM-red wine, DRM-coffee, and HCM-cola subgroups. In contrast, CME generally showed lower adhesion values after beverage exposure compared with the microhybrid composites. These findings suggest that certain material-beverage combinations may be more susceptible to discoloration or bacterial adhesion than would be expected from the main effects of material type or beverage type alone. Therefore, the interaction findings provide a more clinically nuanced interpretation, indicating that the biological and esthetic performance of RCs may depend on the combined effect of material composition and beverage exposure.

An ideal restorative material should have a smooth surface to minimize plaque accumulation and external staining [2]. Furthermore, beverages such as cola, coffee, and wine, which are frequently consumed and have varying pH values, can negatively affect the surface properties of restorative materials due to their acidity and colorants [2, 6, 26]. The current study’s surface roughness findings showed that microhybrid RCs immersed in various beverages exhibited higher surface roughness than nanohybrid RCs with smaller filler particles, consistent with reports that filler particle size affects surface properties [13, 20]. Therefore, the second null hypothesis, that there would be no statistically significant differences between microhybrid and nanohybrid resin composites after beverage immersion in terms of surface roughness and that beverage exposure would not significantly affect surface roughness, was rejected. However, surface roughness is affected not only by filler particle size but also by the type and amount of filler and the structural properties of the organic matrix [2, 6, 11, 26]. These findings indicate that the surface properties of RCs are determined by multifactorial interactions. Previous studies have reported that variations in material composition, including filler type, size, and distribution, may influence the physicochemical and biological properties of resin composites [33–37].

Surface roughness analysis revealed that the highest values were in samples immersed in cola, the beverage group with the lowest pH, while the lowest values were in the distilled water control group, with the highest pH. The absence of a significant RC × beverage interaction for Ra suggests that beverage-induced surface roughness changes were relatively consistent across the tested RC materials. This finding indicates that surface degradation may have occurred through a more material-independent mechanism, in contrast to discoloration and bacterial adhesion, which were modulated by material-beverage interactions. Nevertheless, all mean Ra values were below 0.2 μm, the critical threshold for plaque retention [6]. This suggests that, although statistically significant differences were observed, the clinical relevance of these differences may be limited. Our findings are consistent with similar studies showing that restorative materials become rougher when exposed to low-pH cycles [6, 26, 38]. This may explain why cola, despite inducing the highest surface roughness due to its low pH and erosive potential, did not produce the greatest discoloration, which is more strongly associated with the presence of chromogenic agents rather than acidity alone.

Bacterial adhesion and biofilm formation in the marginal areas of RC restorations can facilitate secondary caries by creating microscopic gaps between tooth tissue and restorative material [18, 20, 39], a leading cause of restoration replacement [18]. Current research focuses on the adhesion and biofilm-forming capacity of *S. mutans*, which can be isolated from almost all carious lesions and from RCs with different chemical compositions [18, 22]. In our study, this bacterium was used, and its adhesion to the tested RCs was analyzed. The findings showed that the lowest median bacterial adhesion was observed in the CME-distilled water subgroup, and the highest in the DRM-cola subgroup. A statistically significant difference in median bacterial adhesion was also found between RCs and beverage groups (*p* < 0.001). The significant RC × beverage interaction further suggests that beverage-related changes in bacterial adhesion were material-dependent, with the microhybrid composites showing greater adhesion increases after exposure to some beverages than the nanohybrid composites. The absence of significant differences among coffee, cola, and red wine as main beverage effects, despite a significant RC × beverage interaction, suggests that the effects of these beverages on bacterial adhesion were composite-specific and may have been obscured when averaged across all RC materials. In other words, the interaction was mainly driven by specific material-beverage combinations, particularly the high adhesion values observed in the DRM-cola, HCM-red wine, DRM-coffee, and HCM-cola subgroups, rather than by a uniform increase caused by one beverage across all materials. This finding indicates that beverage exposure may influence bacterial adhesion through mechanisms that depend on material-specific surface characteristics, such as filler morphology, resin matrix composition, surface roughness, and pellicle-mediated bacterial attachment. Therefore, the third null hypothesis, that there would be no statistically significant differences between microhybrid and nanohybrid resin composites after beverage immersion in terms of bacterial adhesion and that beverage exposure would not significantly affect bacterial adhesion, was rejected. Although statistically significant differences were observed, their clinical relevance should be interpreted with caution.

Previous studies have reported that microhybrid RCs can lead to higher levels of bacterial adhesion than nanohybrids [20, 40], while others have reported no statistically significant difference between the two types of RCs [41]. According to our study results, bacterial adhesion to microhybrid RCs HCM and DRM was significantly higher than that to nanohybrid CME and DRN (*p* < 0.001).

The literature reports that filler content, particle size, and shape affect bacterial adhesion to the RC surface [18]. Some studies have reported that biofilm formation is lower in RCs with higher filler content [18, 42]. In our study, although DRN (83.5%) had a lower median than HCM (79%) and DRM (75%), it had higher values than CME (78%), another RC, contrary to the literature. This discrepancy may be attributed to differences in the chemical composition of the resin matrix, surface energy characteristics, potential variations in polymerization efficiency, and related surface properties such as hydrophobicity and chemical heterogeneity, all of which may collectively influence bacterial adhesion.

Some studies reported a positive correlation between surface roughness and bacterial adhesion [20, 43], whereas others found no significant correlation [22]. Aykent et al. [43] examined the surface roughness and bacterial adhesion of indirect RC, direct RC, and ceramic materials and found a positive correlation among these. In that study, all surface roughness values exceeded the critical threshold of 0.2 μm. In our study, positive correlations between bacterial adhesion and surface roughness were observed only in the DRN-distilled water, DRM-coffee, DRM-cola, DRM-red wine, CME-coffee, CME-cola, and CME-red wine subgroups. The presence of correlations only in selected subgroups may be attributed to the multifactorial nature of bacterial adhesion, which is not solely dependent on surface roughness. While surface roughness may influence bacterial adhesion, other factors such as surface energy, chemical composition, and environmental conditions also play a role, which may explain the lack of a consistent correlation across all subgroups.

This study has several limitations. The in vitro design, based on static immersion and a single-species *S. mutans* adhesion model, does not fully replicate the dynamic oral environment, including salivary flow, pH fluctuations, thermal cycling, mechanical forces, and multispecies biofilm interactions. The use of saliva from a single donor represents an important biological limitation of this study. Although this approach was chosen to standardize pellicle formation and reduce inter-individual variability, salivary protein composition, including proline-rich proteins, mucins, statherin, and histatins, varies substantially among individuals and directly influences acquired pellicle composition and bacterial adhesion. Therefore, the pellicle formed in this study may not represent the range of acquired pellicles encountered under clinical conditions, and the bacterial adhesion findings cannot be fully generalized to broader patient populations. Future studies should consider using saliva from multiple donors or standardized artificial saliva formulations, such as Fusayama’s artificial saliva or Glandosane, to better evaluate the influence of pellicle variability on bacterial adhesion. Although a 1-hour saliva incubation period has been used in previous resin composite bacterial adhesion protocols, it may not fully reflect the compositional maturation of the acquired pellicle under in vivo conditions. In addition, continuous immersion does not simulate the intermittent and time-dependent exposure patterns of real beverage consumption. The correlation analyses were performed within relatively small subgroups (*n* = 12), which may increase the risk of type I error and should therefore be interpreted with caution. Moreover, although the a priori sample size calculation was based on a parametric two-way ANOVA assumption, the CFU data were non-normally distributed and were analyzed using two-way robust ANOVA. Therefore, the statistical power for the CFU outcome with *n* = 12 per subgroup may be limited. Furthermore, the 12-day immersion period should be considered an approximation rather than a validated simulation of long-term clinical exposure. The pH of the solutions was not monitored during the experimental period, which may limit the chemical interpretation of the results. SEM analysis was limited to one specimen per subgroup; therefore, the findings should be interpreted as illustrative rather than generalizable. Finally, only four composite resins were evaluated, including two from the same manufacturer, which may limit the representativeness of the findings.

## Conclusions

It was observed that beverages with different pH values (coffee, cola, and red wine) significantly affected discoloration, surface roughness, and bacterial adhesion of the tested RCs, including nanohybrid and microhybrid types. The highest ∆*E*_00_ was observed with red wine, and there was no statistically significant difference between red wine and coffee. The surface roughness for DRN was not statistically significantly different from that of CME; however, it was significantly different from that of the other RCs. Microhybrid RCs had higher median bacterial adhesion than the other RCs. Within the limitations of this in vitro study, beverage exposure significantly influenced discoloration, surface roughness, and bacterial adhesion of the tested RCs. These findings are limited to the materials tested in this study and should not be generalized to all RC types. However, these results may be considered in the clinical selection and long-term evaluation of restorative materials.

## Data Availability

The data that support the findings of this study are available on request from the corresponding author. The data are not publicly available dueto privacy or ethical restrictions.

## Abbreviations

CFU: Colony-forming unit
RC: Resin composite
*S. mutans*: *Streptococcus mutans*
SEM: Scanning electron microscopy
LED: Light-emitting diode
PBS: Phosphate-buffered saline
BHI: Brain heart infusion broth
MSB: Mitis salivarius bacitracin agar
DRM: Dr. Müller Rhein
HCM: Herculite Classic
DRN: Dr. Müller Kaiser
CME: Clearfil Majesty Esthetics
Δ*E*_00_: Color difference (CIEDE2000)
Ra: Surface roughness average
